# Treatment of Spinal Metastases with Epidural Cord Compression through Corpectomy and Reconstruction via the Traditional Open Approach versus the Mini-Open Approach: A Multicenter Retrospective Study

**DOI:** 10.1155/2019/7904740

**Published:** 2019-05-02

**Authors:** Xi Zhou, Haomin Cui, Yu He, Guixing Qiu, Dongsheng Zhou, Yong Liu

**Affiliations:** ^1^Department of Orthopaedic Surgery, Peking Union Medical College Hospital, Chinese Academy of Medical Sciences & Peking Union Medical College, No. 1 Shuai Fu Yuan, Wang Fu Jing Street, Beijing 100730, China; ^2^Department of Orthopedic Surgery, Shanghai Jiao Tong University Affiliated Sixth People's Hospital, 600 Yishan Road, Shanghai 200233, China; ^3^Department of Plastic Surgery, Plastic Surgery Hospital, Chinese Academy of Medical Sciences & Peking Union Medical College, No. 33 Badachu Road, Beijing 100144, China; ^4^Department of Orthopaedic Surgery, Shandong Provincial Hospital Affiliated to Shandong University, 324 Jingwu Road, Ji'nan, Shandong 250021, China

## Abstract

Patients with metastatic epidural spinal cord compression (MESCC) often need surgical intervention due to pain, neurological deficits, and spinal instability. Spinal disease is commonly treated via the minimally invasive mini-open approach. However, few studies have evaluated MESCC treatment via mini-open approach. The present study compared the traditional open approach versus the mini-open approach for thoracolumbar MESCC. A cohort of 209 consecutive patients who were diagnosed with thoracolumbar metastases and underwent corpectomy and polymethylmethacrylate reconstruction from 2010 to 2016 was retrospectively identified. Traditional open surgery was performed in 113 patients (open group; mean age 57.7 years), while 96 patients underwent mini-open surgery (mini-open group; mean age 54.3 years). Patients were followed up for 24 months or until death. The baseline characteristics of both groups were similar. The most common origin of the primary lesion was the lung (37.3%), hematological system (22.0%), and kidney (15.8%). Surgery effectively achieved pain relief, restored neurological function, and improved quality of life in both groups. The mini-open group was superior to the open group regarding estimated blood loss, blood transfusion, hospital stay, complications, and pain score. While the mini-open group had a longer operation time than the open group, the two groups had similar improvements in the Frankel grade and Karnofsky functional score. The 30-day mortality rate tended to be higher in the open group (5.3%) than the mini-open group (2.1%) without significance. The 24-month survival rate was similar in both groups (26.5% versus 26.0%). In conclusion, surgery improved pain, function, and quality of life in patients with MESCC. The mini-open approach resulted in less estimated blood loos, less blood transfusion, and shorter hospitalization than the traditional open approach, while both methods had similar mortality and morbidity rates. Thus, the mini-open approach may be more beneficial than the traditional approach for MESCC.

## 1. Introduction

Spinal metastasis accounts for approximately 60% of all osseous metastatic disease, and occult spinal disease is present in at least 25% of patients who die as a result of malignant tumors [1–3]. Most studies indicate that malignant tumors most commonly originate in the lung, breast, prostate, kidney, and hematologic system [1, 4, 5]. Hematogenous spread is responsible for over 85% of cases of metastatic epidural spinal cord compression (MESCC) with vertebral collapse and compression [4]. MESCC is a devastating consequence of spinal metastases, and is an oncologic emergency that requires rapid diagnosis and treatment. MESCC causes marked impairments in quality of life due to pain and neurological dysfunction [1, 4, 6].

The appropriate treatment of MESCC is a huge challenge that requires multidisciplinary collaboration [7]. The goals of MESCC treatment are to improve quality of life, maintain or improve neurological function, and relieve pain through spinal cord decompression, spinal stability, and local tumor control [1, 4, 6]. The most common therapies used to treat MESCC are surgery, radiotherapy, or a combination of these two methods [1, 4, 5, 8]. Radiotherapy is effective and widely used. However, many patients require surgery due to neurological deficits, pain, and vertebral collapse. The surgical indications include MSECC with relative radio-resistance, tumor progression following radiation therapy, or spinal instability [1–6]. MESCC is surgically treated via a variety of methods and approaches. The ideal excision method is en bloc resection [9]. However, the complex anatomy of the spine makes this radical procedure extremely difficult or even impossible [9, 10]. Thus, palliative debulking methods are preferred for patients with a short life expectancy, as these methods are simpler and have lower morbidity rates [1, 4, 9, 10].

The single posterior approach is considered the standard approach to the thoracolumbar spine, and its benefits over the anterior approach include excellent exposure, direct extension from the vertebral body to the posterior elements, and bilateral dura holes control [11, 12]. However, traditional open surgery involves large exposure, extensive muscular and fascial dissection, and a relatively large amount of bleeding; these complications may be overcome by minimally invasive surgery (MIS). In MESCC, percutaneous or small skin incision MIS could be used to achieve adequate corpectomy and decompression difficultly; moreover, the unclear anatomic landmarks in MESCC make the operation even impossible. The mini-open approach seems to achieve a balance between lessening the surgical trauma and achieving better corpectomy and decompression. However, the application of mini-open surgery has been concentrated on the treatment of degenerative spinal disorders, and has rarely been evaluated for MESCC. The present study compared the traditional open approach and the mini-open approach for the treatment of thoracolumbar MESCC via corpectomy and polymethylmethacrylate (PMMA) reconstruction.

## 2. Materials and Methods

The present study was approved by the Institutional Review Board of Peking Union Medical College Hospital.

### 2.1. Patients

We retrospectively reviewed the medical records of 308 patients diagnosed with MESCC between 2010 and 2016 at Peking Union Medical College Hospital, Shanghai Jiaotong University Affiliated Sixth People's Hospital, and Shandong Provincial Hospital Affiliated to Shandong University. The diagnosis of MESCC was confirmed in accordance with clinical, radiological, and pathological criteria. Surgery was performed in 226 patients. Of these, 17 patients did not complete follow-up. Thus, a final total of 209 patients (86 females and 123 males) with a mean age of 56.2 years were included in the present study. Multiple metastases to visceral organs and/or bones were present in 135 patients at the time of surgery.

The most common symptom was pain in 176 patients (84.2%), motor or sensory deficits in 124 (59.3%), and paraparesis or paraplegia in 21 (10.0%). The preoperative Frankel grading of motor and sensory neurological deficits was grade A (complete paraplegia) in 1.0% of patients, B (no motor function) in 3.8%, C (motor function present, but useless) in 18.7%, D (slight motor function deficit) in 25.8%, and E (no motor deficit) in 50.7%.

### 2.2. Surgery

All patients received multidisciplinary evaluation by an oncologist, chemoradiologist, protopathy expert, and orthopedic expert and had a satisfactory general condition and a life expectancy of more than 3 months. Surgical indications included MSECC with relative radio-resistance, tumor progression after chemoradiotherapy, intractable pain resistant to other methods, neurological deficits, or spinal instability. However, posterior soft tissue invaded by tumor, posterior cortical destruction (especially for pedicle), severe spinal deformities caused by tumor, and pedicle dysplasia were considered as contraindications.

**Figure 1:**
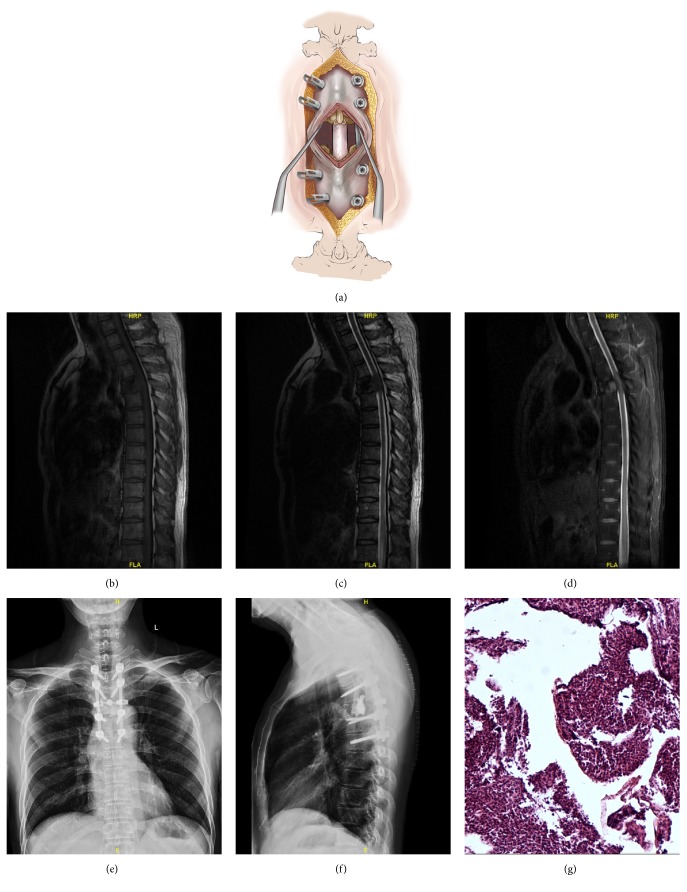
Case example of a 52-year-old male with severe thoracic spinal stenosis and myelopathy due to myeloma involvement of T4. Illustrations showing the mini-open approach, demonstrating the little soft-tissue dissection (a). The thoracic magnetic resonance imaging (b-d) shows metastatic epidural spinal cord compression with tumor involvement of the T4 vertebral body. The patient underwent laminectomy, corpectomy, tumor resection, and polymethylmethacrylate reconstruction of T4, plus pedicle screw instrumentation of T2-T6. Postoperative anteroposterior and lateral radiography shows stable reconstruction (e-f). Pathological examination shows that the lesion was consistent with plasma cell myeloma (g).

The mini-open approach for transpedicular corpectomy and pedicle fixation has been described previously [11, 13]. In brief, the skin was opened along the median line, while the fascia was preserved. A percutaneous pedicle screw fixation system was applied. After fixation, the fascia and muscle were opened at the level of the corpectomy, and the posterior spinal elements were exposed (Figure 1). Transpedicular corpectomy was then performed in a routine manner. The specific resection region depended on the extent of the tumor. The discs and posterior longitudinal ligament were often involved. A trap-door rib head osteotomy was performed when necessary. After corpectomy, PMMA was used for the reconstruction of the vertebral body.

**Figure 2:**
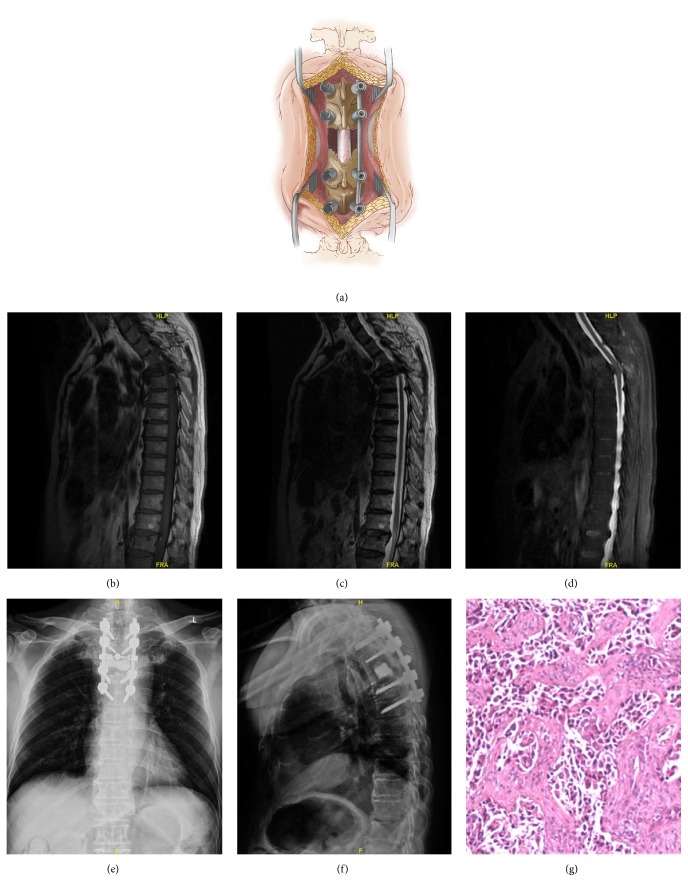
Case example of a 62-year-old male with metastatic lung cancer and walking difficulty. Illustrations showing the open approach, demonstrating the fascia and muscle opened at every level of the exposed region (a). Metastatic epidural spinal cord compression of T4 is seen on thoracic magnetic resonance imaging (b-d). The patient underwent laminectomy, corpectomy, tumor resection, and polymethylmethacrylate reconstruction of T4, plus pedicle screw instrumentation of T2-T6. Postoperative imaging shows stable reconstruction (e-f). Pathological examination shows that the lesion was consistent with adenocarcinoma of the lung (g).

Traditional open surgery for pedicle fixation, corpectomy, and PMMA reconstruction was performed in accordance with routine methods. The fascia and muscle were opened at every level of the exposed region (Figure 2).

Percutaneous vertebroplasty is considered an effective method with which to relieve pain and stabilize the spine [1]; thus, this method was used to treat other metastatic vertebrae without epidural cord compression when necessary. Intervertebral bone grafting was rarely performed. The operative region was washed with cisplatin solution before wound closure.

Postoperative management was tailored to each individual patient, and comprised antibiotics, steroid administration, and deep venous thrombosis prophylaxis. Early activity and physiotherapy were begun as soon as possible. Progressive mobilization of sitting, ambulation, and walking was performed gradually. An external orthosis was used during the first month postoperatively.

### 2.3. Outcomes

The operation-related outcomes included operative time, estimated blood loss (EBL), transfusions of red blood cells (RBC) and fresh frozen plasma (FFP), hospital stay, and complications. The functional outcomes included the visual analogue score (VAS) for pain, Frankel grade, and Karnofsky functional score. Patients were followed up for 2 years or until death.

### 2.4. Statistical Analysis

Statistical analyses were performed with SPSS software (version 19.0; SPSS Inc., Chicago, IL). Differences between groups were compared via one-way analysis of variance, the Kruskal-Wallis test, and the Student's t-test as appropriate. The Kaplan-Meier method was used to analyze the survival rate. The level of statistical significance was defined as p<0.05.

## 3. Results

**Figure 3:**
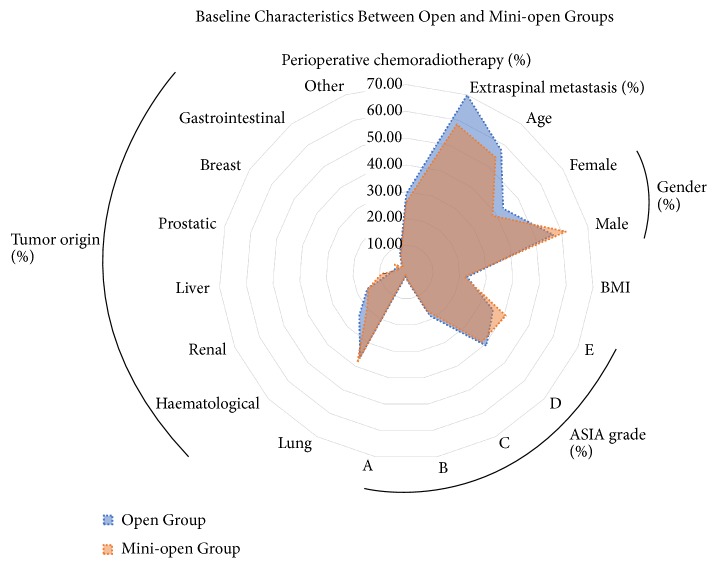
The patient demographics and characteristics. The baseline characteristics of the open and mini-open groups were similar, with no significant intergroup differences in age, tumor origin, extraspinal metastasis, or ASA grade. The mean BMI significantly differed between the two groups (p=0.005).

The patient demographics and characteristics are summarized in Table 1 and Figure 3. The traditional open approach was used in 49 females and 64 males with a mean age of 57.7 years (open group), while the mini-open approach was used in 37 females and 59 males with a mean age of 54.3 years (mini-open group). The baseline characteristics of the two groups were similar, with no significant intergroup differences in age, tumor origin, extraspinal metastasis, or ASA grade. The most common origin of the primary lesion was the lung (37.3%), hematological system (22.0%), and kidney (15.8%). Perioperative radiotherapy or chemotherapy was performed in 33 (29.2%) patients in the open group, and 25 (26.0%) in the mini-open group. The average hospital stays in the open and mini-open groups were 21.6 days and 15.3 days, respectively.

### 3.1. Surgical Outcomes

**Figure 4:**
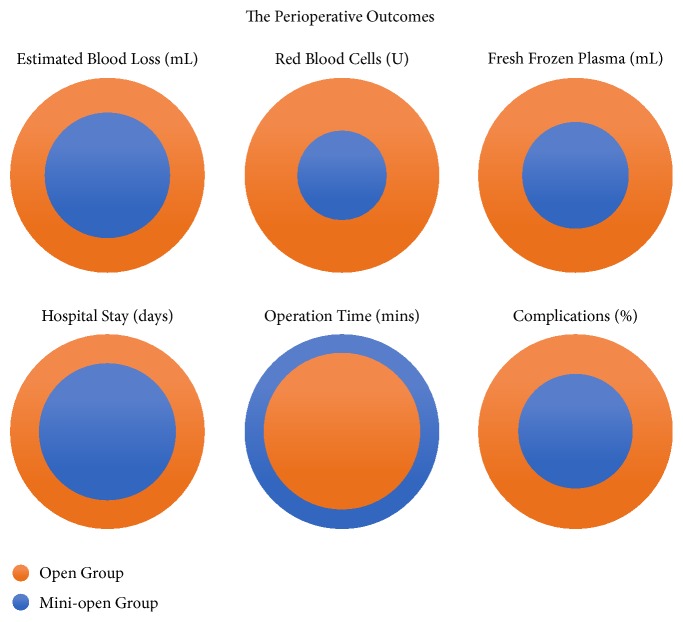
The perioperative outcomes. The open group had a significantly greater average estimated blood loss, blood transfusions of red blood cells and fresh frozen plasma, hospital stay, and complications than the mini-open group (p < 0.05). The average operation time in the open group was significantly shorter than that in the mini-open group (p < 0.001).

The perioperative outcomes are shown in Table 2 and Figure 4. There was no significant difference in the number of corpectomy and reconstruction procedures performed in each patient in the open group (1.9) and the mini-open group (1.8). The average operation time in the open group (225.2 min) was significantly shorter than that in the mini-open group (276.7 min; p < 0.001). The open group had a significantly greater average EBL (1,534.5 ml) than the mini-open group (1,007.3 ml; p < 0.001). Blood transfusion was administered to a significantly greater proportion of patients in the open group (93.8%; with a mean of 5.2 U of RBC and 587.6 ml of FFP) compared with the mini-open group (89.6%; with a mean of 2.4 U of RBC and 327.1 ml of FFP; p < 0.001).

### 3.2. Symptom Relief and Functional Outcomes

**Figure 5:**
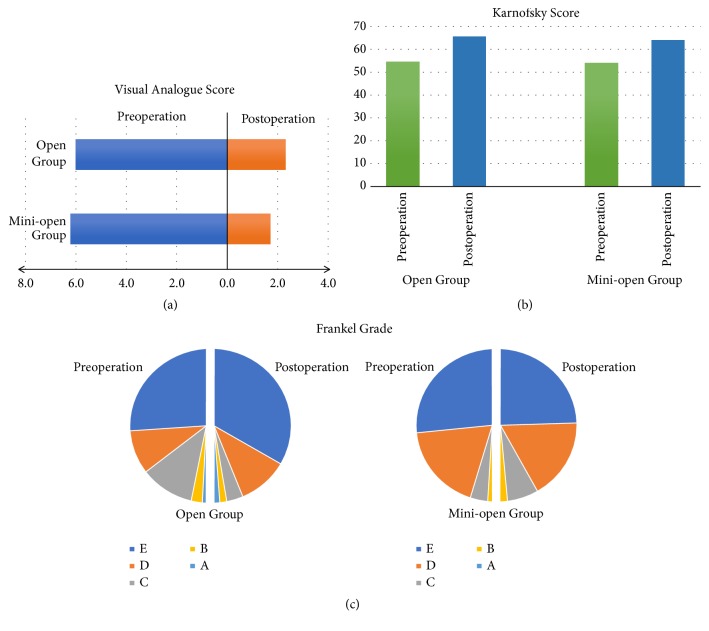
The postoperative outcomes of the visual analogue score, Frankel grade, and Karnofsky scores. The average postoperative decreases in the visual analogue score were 3.7 and 4.5 points in the open and mini-open groups (a). The Karnofsky scores (b) and Frankel grade (c) improved postoperatively in both groups.

The postoperative outcomes are summarized in Table 3. The pain was relieved in 92.8% of all patients. The VAS was improved in 103 (91.2%) patients in the open group, and 91 (94.8%) in the mini-open group. The average postoperative decreases in the VAS were 3.7 and 4.5 points in the open and mini-open groups (Figure 5(a)), respectively. The neurological deficit was fully resolved postoperatively in 108 patients. The Frankel grade improved postoperatively in both groups, and improved walking ability was observed in 148 patients (Figure 5(b)). The respective Karnofsky scores in the open and mini-open groups improved from 54.6 and 54.0 preoperatively to 65.5 and 63.8 postoperatively (Figure 5(c)). The quality of life was improved in 60.2% and 62.5% of patients in the open and mini-open groups, respectively.

### 3.3. Complications

Overall, postoperative complications occurred in 27 patients (Table 2). The most common complications were wound infection/breakdown (17.1%), acute neurological aggravation (14.6%), and symptomatic local tumor recurrence (14.6%). In the open group, 18 patients experienced 30 complications including wound infection/breakdown, pedicle screw misplacement, acute neurological aggravation, and symptomatic local tumor recurrence. The mini-open group most frequently experienced dural tear, symptomatic local tumor recurrence, and wound infection/breakdown. Thirteen patients required reoperation for debridement of infection, adjustment of pedicle screw positioning, and tumor recurrence.

### 3.4. Survival Rates

**Figure 6:**
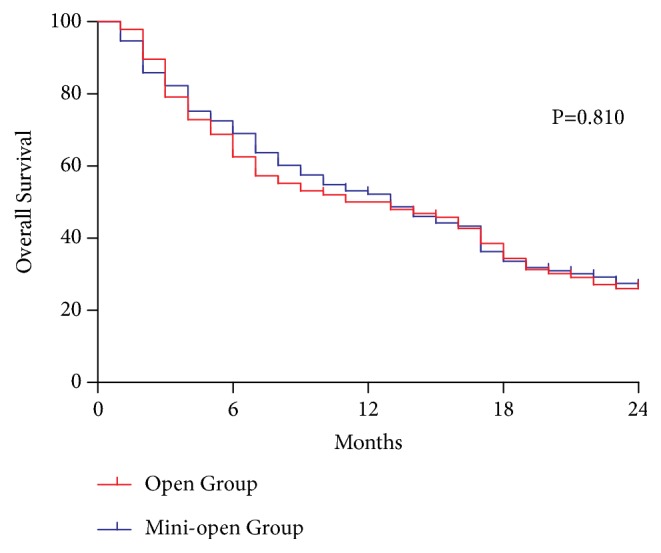
The 24-month survival rates. The 24-month survival rates were similar in the open (26.5%) and mini-open (26.0%) groups (p=0.810).

The 30-day mortality rate of the open group (5.3%) tended to be higher than that of the mini-open group (2.1%); however, this intergroup difference was not significant (p=0.226). The 24-month survival rates were similar in the open and mini-open groups (26.5% and 26.0%, respectively; p=0.810; Figure 6).

## 4. Discussion

Bone is the third most common site for metastases (following the liver and lungs), and most bone metastases are located in the spine. As many as 10% of patients with spinal metastases develop MESCC and experience neurological deficits, pain, and vertebral collapse. MIS is a suitable method that improves patient quality of life; moreover, MIS minimizes the morbidity and shortens the recovery time compared with open surgery. The present study compared the traditional open approach versus the mini-open approach for the treatment of thoracolumbar MESCC via corpectomy and PMMA reconstruction.

The application of MIS via the single posterior approach for the treatment of MESCC still lacks adequate evaluation; however, a few previous studies indicate that MIS is a promising prospect for MESCC treatment. One previous study that evaluated a consecutive cohort who underwent thoracic transpedicular corpectomies for spinal metastases via the mini-open approach (n=21) or the open approach (n=28) reported that the mini-open approach was associated with less blood loss and shorter hospital stay compared with open surgery [11]. Another prospective study reported good outcomes for 10 patients with spinal metastases who underwent corpectomy and percutaneous instrumentation by MIS, suggesting that MIS treatment of thoracolumbar spinal metastases was a safe and effective palliative method that could limit morbidity and preserve quality of life [14]. Other studies have reported similar findings for MIS treatment of spinal metastases, with less muscle injury, less blood loss, shorter hospital stay, and lower rates of infection compared with open surgery [15–18].

In our study, patients with thoracolumbar MESCC who underwent corpectomy and PMMA reconstruction via the mini-open approach achieved more benefits than those who underwent surgery via the traditional open approach. In particular, the mini-open group had less EBL, less blood transfusion, and shorter hospitalization than the open group. Blood loss and blood transfusion are affected by many factors, including surgical techniques, tumor characteristics, and general condition. Nevertheless, compared with the mini-open approach, the wider fascial and muscle exposure in the open approach is probably the reason for the greater amount of bleeding and greater requirement for intra- and postoperative blood transfusions. Several studies have reported that greater amounts of perioperative blood transfusion in patients with cancer are related to increased 30-day mortality postoperatively and more unexpected complications [19, 20]. Thus, the relatively reduced blood transfusion requirement in mini-open surgery may achieve additional benefits.

The operation time was significantly prolonged in the mini-open group compared with the open group, which was probably due to the longer preparation time and increased fluoroscopy time. However, although increased operation time may lead to more operation-related complications, the present study found no increase in operative risk in the mini-open group compared with the open group; the benefits of the mini-open approach may outweigh the risks associated with the longer operative time.

The hospital stay is also affected by many factors, including comorbidities, complications, and other unexpected reasons. The present study found that the mini-open group had a shorter duration of hospitalization than the open group. A shorter hospital stay may avoid hospital-related complications, directly represent the occurrence of less postoperative complications, and indirectly reflect faster recovery. In our study, the incidence of postoperative complications in the mini-open group was 9.4%, which tended to be lower than that in the open group (15.9%), although this difference did not reach statistical significance. Furthermore, the rate of reoperation was higher after open surgery than mini-open surgery, which may play a role in speeding up the recovery to shorten the duration of hospitalization. Previous studies have reported that posterior-based corpectomy with a large incision induces extensive stripping of the paraspinal muscles, which is related to high morbidity and complication rates and extended recovery time [11, 21]. In addition, we believe that less wound pain and better patient mobility resulting from the relatively lesser fascial and muscular dissection in the mini-open approach are also key factors in improving postoperative recovery.

The present study found that mini-open surgery positively affected pain relief, recovery of neurological deficits, improvement of quality of life, and survival rate. After discharge from hospital, pain relief was achieved by 92.8% of all patients, with a significantly greater decrease in the VAS in the mini-open group than in the open group. The relatively lower postoperative VAS after mini-open surgery compared with open surgery may be explained by the lesser wound pain caused by the lesser dissection of the fascia and muscles. The neurological function and quality of life was similarly improved postoperatively in both groups. This result is in agreeance with previous studies that reported that almost half of the patients with neurological deficits achieve recovery after surgical intervention [6]. A prospective study including 118 patients with spinal metastases suggested that surgery improves pain, neurological deficits, sphincteric dysfunction, and ambulatory status [6]. Approximately half of the patients in this previous study achieved complete resolution of pain and neurological deficits, and the 12-month mortality rate was 48%, which was similar to our results. Furthermore, the Karnofsky performance status in the present study indicated that surgery improved the quality of life to a similar extent in both groups.

For patients with spinal metastases, any beneficial effect that may be gained from new surgical technology should be weighed against potential complications and morbidity. In the present cohort, the mini-open group had no increases in complication and morbidity rates compared with the open group. The overall complication rate in our study was 12.9%, which is in accordance with previous reports ranging from 5 to 30% [1, 4, 6, 10]. The 24-month mortality rate was similar between the two groups, while the 30-day mortality rate tended to be lower in the mini-open group than the open group (but without statistical significance). The greater blood loss and increased transfusion requirements may be attributed to the greater 30-day mortality in the open group than in the mini-open group. Moreover, the mortality rate was largely dependent on tumor characteristics, which supports the results of other studies [2].

The present study had some limitations. The major shortcomings are related to the inherent nature of retrospective studies; thus, selection bias and recall bias may affect the accuracy of the present findings. Furthermore, the mean BMI significantly differed between the two groups, because there was a tendency for surgeons to perform MIS in patients with a lower BMI, which may affect the validity of the present results. In addition, there may have been interinstitutional differences in techniques and protocols; however, there was little difference in the procedures performed by the different surgical teams. Finally, it was difficult to avoid confounder biases; however, the baseline characteristics of the two groups were well matched.

## 5. Conclusions

In conclusion, surgical intervention improves pain, function, and quality of life in patients with MESCC. The application of MIS technology has been increasing in MESCC surgery over the past decade. The mini-open approach was associated with less blood loss, less blood transfusion, and shorter hospitalization than the traditional open approach. As the complications and morbidity rates were unaffected by the surgical approach in the present study, patients may benefit more from mini-open surgery compared with open surgery.

## Figures and Tables

**Table 1:** Patients' demographics and characteristics.

Patients		Open Group	Mini-open Group	p Value
		(n = 113)	(n = 96)	
Gender				0.480
	Female	49 (43.3%)	37 (38.5%)	
	Male	64 (56.6%)	59 (61.5%)	
Age		57.7	54.3	0.074
BMI		22.8	21.7	0.005
ASIA grade				0.419
	E	40 (35.4%)	39 (40.6%)	
	D	46 (40.7%)	37 (38.5%)	
	C	21 (18.6%)	16 (16.7)	
	B	4 (3.5%)	3 (3.1%)	
	A	2 (1.8%)	1 (1.0%)	
Tumor origin				0.952
	Lung	41 (36.3%)	37(38.5%)	
	Haematological	27 (23.9%)	19 (19.8%)	
	Renal	18 (15.9%)	15 (15.6%)	
	Liver	7 (6.2%)	9 (9.4%)	
	Prostatic	5 (4.4%)	3 (3.1%)	
	Breast	3 (2.7%)	5 (5.2%)	
	Gastrointestinal	4 (3.5%)	2 (2.1%)	
	Other	8 (7.1%)	6 (6.3%)	
Perioperative chemoradiotherapy		33 (29.2%)	25 (26.0%)	0.611
Extraspinal metastasis		79 (69.9%)	56 (58.3%)	0.081

BMI, Body Mass Index.

**Table 2:** The perioperative outcomes.

Perioperative Outcomes		Open Group	Mini-open Group	p Value
		(n = 113)	(n = 96)	
Number of corpectomy and reconstruction		1.9	1.8	0.800
EBL (ml)		1534.5	1007.3	<0.001
Blood transfusion				
	Number of Patients	106 (93.8%)	86 (89.6%)	0.266
	RBC (U)	5.2	2.4	<0.001
	FFP (ml)	587.6	327.1	<0.001
Operation time (min)		225.2	276.7	<0.001
Length of Hospitalization (days)		21.6	15.3	0.006
Complications				0.390
	Number of Patients	18 (15.9%)	9 (9.4%)	0.159
	Wound infection/breakdown	5 (16.7%)	2 (18.2%)	
	Pedicle screw misplacement	5 (16.7%)	0 (0.0%)	
	Acute neurological aggravation	4 (13.3%)	2 (18.2%)	
	Symptomatic local tumour recurrence	4 (13.3%)	2 (18.2%)	
	Dural tear	3 (10.0%)	2 (18.2%)	
	Spinal shock	2 (6.7%)	0 (0.0%)	
	Infectious shock	2 (6.7%)	1 (9.1%)	
	Deep vein thrombosis	2 (6.7%)	0 (0.0%)	
	Pleural tear	1 (3.3%)	0 (0.0%)	
	Bone cement misplacement	1 (3.3%)	0 (0.0%)	
	Cerebral infarction	1 (3.3%)	0 (0.0%)	
	Respiratory	0 (0.0%)	1 (9.1%)	
	Cardiovascular	0 (0.0%)	1 (9.1%)	
Re-operation		9 (8.0%)	4 (4.2%)	0.390
30-day Mortality Rate		5.3%	2.1%	0.226

EBL, estimated blood loss; RBC, red blood cells; FFP, fresh frozen plasma.

**Table 3:** Symptoms relief and functional outcomes.

Scores		Preoperation		Postoperation		p Value
		Open Group	Mini-open Group	Open Group	Mini-open Group	
VAS		6.0	6.2	2.3	1.7	<0.001
Frankel Grade						
	E	59 (52.2%)	47 (49.0%)	75 (66.4%)	51 (53.1%)	<0.001
	D	21 (18.6%)	33 (34.4%)	24 (21.2%)	36 (37.5%)	
	C	26 (23.0%)	13 (13.5%)	8 (7.1%)	7 (7.3%)	
	B	5 (4.4%)	3 (3.1%)	3 (2.7%)	2 (2.1%)	
	A	2 (1.8%)	0 (0.0%)	3 (2.7%)	0 (0.0%)	
Karnofsky Score		54.6	54	65.5	63.8	<0.001

VAS, visual analogue score.

## Data Availability

The data used to support the findings of this study are available from the corresponding author upon request.

## References

[1] Spratt D. E., Beeler W. H., de Moraes F. Y. (2017). An integrated multidisciplinary algorithm for the management of spinal metastases: an International Spine Oncology Consortium report. *The Lancet Oncology*.

[2] Molina C., Goodwin C. R., Abu-Bonsrah N., Elder B. D., De la Garza Ramos R., Sciubba D. M. (2016). Posterior approaches for symptomatic metastatic spinal cord compression. *Neurosurgical Focus*.

[3] Street J., Fisher C., Sparkes J. (2007). Single-stage posterolateral vertebrectomy for the management of metastatic disease of the thoracic and lumbar spine: a prospective study of an evolving surgical technique. *Journal of Spinal Disorders & Techniques*.

[4] Al-Qurainy R., Collis E. (2016). Metastatic spinal cord compression: diagnosis and management. *BMJ*.

[5] Patel D. A., Campian J. L. (2017). Diagnostic and therapeutic strategies for patients with malignant epidural spinal cord compression. *Current Treatment Options in Oncology*.

[6] Quan G. M. Y., Vital J.-M., Aurouer N. (2011). Surgery improves pain, function and quality of life in patients with spinal metastases: a prospective study on 118 patients. *European Spine Journal*.

[7] Arana E., Kovacs F. M., Royuela A., Asenjo B., Pérez-Ramírez Ú., Zamora J. (2016). Back pain research network task force for the improvement of inter-disciplinary management of spinal, "agreement in metastatic spinal cord compression". *Journal of the National Comprehensive Cancer Network*.

[8] Laufer I., Iorgulescu J. B., Chapman T. (2013). Local disease control for spinal metastases following "separation surgery" and adjuvant hypofractionated or high-dose single-fraction stereotactic radiosurgery: outcome analysis in 186 patients. *Journal of Neurosurgery: Spine*.

[9] Boriani S., Gasbarrini A., Bandiera S., Ghermandi R., Lador R. (2016). Predictors for surgical complications of en bloc resections in the spine: review of 220 cases treated by the same team. *European Spine Journal*.

[10] Delank K., Wendtner C., Eich H. T., Eysel P. (2011). The treatment of spinal metastases. *Deutsches Aerzteblatt Online*.

[11] Lau D., Chou D. (2015). Posterior thoracic corpectomy with cage reconstruction for metastatic spinal tumors: comparing the mini-open approach to the open approach. *Journal of Neurosurgery: Spine*.

[12] Shen F. H., Marks I., Shaffrey C., Ouellet J., Arlet V. (2008). The use of an expandable cage for corpectomy reconstruction of vertebral body tumors through a posterior extracavitary approach: a multicenter consecutive case series of prospectively followed patients. *The Spine Journal*.

[13] Chou D., Lu D. C. (2011). Mini-open transpedicular corpectomies with expandable cage reconstruction: technical note. *Journal of Neurosurgery: Spine*.

[14] Zairi F., Arikat A., Allaoui M., Marinho P., Assaker R. (2012). Minimally invasive decompression and stabilization for the management of thoracolumbar spine metastasis. *Journal of Neurosurgery: Spine*.

[15] Dong Y. L., Jung T.-G., Lee S.-H. (2008). Single-level instrumented mini-open transforaminal lumbar interbody fusion in elderly patients. *Journal of Neurosurgery: Spine*.

[16] Tsutsumimoto T., Shimogata M., Ohta H., Misawa H. (2009). Mini-open versus conventional open posterior lumbar interbody fusion for the treatment of lumbar degenerative spondylolisthesis: comparison of paraspinal muscle damage and slip reduction. *The Spine Journal*.

[17] Dhall S. S., Wang M. Y., Mummaneni P. V. (2008). Clinical and radiographic comparison of mini-open transforaminal lumbar interbody fusion with open transforaminal lumbar interbody fusion in 42 patients with long-term follow-up: clinical article. *Journal of Neurosurgery: Spine*.

[18] Gerszten P. C., Monaco E. A. (2009). Complete percutaneous treatment of vertebral body tumors causing spinal canal compromise using a transpedicular cavitation, cement augmentation, and radiosurgical technique. *Neurosurgical Focus*.

[19] Al-Refaie W. B., Parsons H. M., Markin A., Abrams J., Habermann E. B. (2012). Blood transfusion and cancer surgery outcomes: a continued reason for concern. *Surgery*.

[20] Xenos E. S., Vargas H. D., Davenport D. L. (2012). Association of blood transfusion and venous thromboembolism after colorectal cancer resection. *Thrombosis Research*.

[21] Di Martino A., Vincenzi B., Denaro L. (2009). 'Internal bracing' surgery in the management of solid tumor metastases of the thoracic and lumbar spine. *Oncology Reports*.

